# Supplementary material for: Patterns of Intron Gain and Loss in Fungi
Source: PLoS Biol. 2004 Nov 30;2(12):e422. doi: 10.1371/journal.pbio.0020422 (PMC532390; doi:10.1371/journal.pbio.0020422)
Supplement: Table S1 — Also available at http://genes.mit.edu/NielsenEtAl/. (4.3 MB ZIP). [file pbio.0020422.st001.zip › NielsenEtAl/html/42.html]

AN1965.1.NCU06970.1.MG07148.1.FG09299.1


```
 CLUSTAL W (1.82) Multiple Sequence Alignments - Introns Inserted


Sequence 1: NCU06970.1	323 aa
Sequence 2: FG09299.1	323 aa
Sequence 3: MG07148.1	345 aa
Sequence 4: AN1965.1	320 aa
Alignment Length: 346 aa
Number Identitical Residues: 205 aa
Alignment Score (without introns) 9089


MG07148.1 	-MSSTSNSIKLLSGNSH2MLLGRLVADR2LGIEIAKTLSLNYSNQETSVTVGESVRDEDV
NCU06970.1	MSGEMANEVKLISGRSH~PELSEKVAKR2LGIEVARTISLNYSNQETSFTVGESVRDEDV
FG09299.1 	MLDQMANEIKLISGSSH~PEISAKVASR2LGIEIANTMSLNYSNRETSVSIGESVRDEDV
AN1965.1  	---MATNSIKLLTGNSH~PELANLVADR2LGIELTKIMVLQYSNQETSVTIGESVRDEDV
          	     :*.:**::* **   :.  **.* ****::. : *:***:***.::*********

MG07148.1 	~FIIQSTMPGDINDG1LMELLIMTHA1CRTASARRITC2VL~PNFP1YA1RQDKKDRSRA
NCU06970.1	~FIIQSTTTGDVNEG~LMELLIAISA~CRTASARRITA~VI2PNFP~YA~RQDKKDKSRA
FG09299.1 	~FILQSTAPGDVNDG~LMELLIMIHA~CRTASARRITA~VI~PSFP~YA~RQDKKDKSRA
AN1965.1  	1FILQSTRPNDINDG~LMELLIMINA~CKTASARRITA~VI~PNFP~YA~RQDKKDKSRA
          	 **:*** ..*:*:* ******   * *:********. *: *.** ** ******:***

MG07148.1 	1PISAKLIA2NMI0QTAGC~NH~VITMDL0HASQ~IQG~FFNIPV~DNLYA0EPSVLRWI
NCU06970.1	~PISARLVA~NML~QTAGA~NH1IVTVDL~HASQ0IQG1FFSVPV1DNLYA~EPSFLRYI
FG09299.1 	~PISAKLIA~NML~QVSGC0NH~VITMDL~HASQ~IQG~FFNVPV~DNLYA~EPSVLRWI
AN1965.1  	~PITAKLMA~NML~QTAGC~NH~VITMDL~HASQ~IQG~FFNVPV~DNLYA~EPSMLKWI
          	 **:*:*:* **: *.:*. ** ::*:** **** *** **.:** ***** ***.*::*

MG07148.1 	RENLPVEKCVIVSPDAGGAKR~ATSIADRLDLGFALIHK2ERPRPNVVGRM~VLVGDVKD
NCU06970.1	RENYKPEDCVIVSPDAGGAKR2ATSIADHLNTGFALIHK~ERPRPNVVGRM0VLVGNVED
FG09299.1 	RENLNVENCVIVSPDAGGAKR~ATSLADRLNTGFALIHK~ERPRPNVVGRM~VLVGDVQD
AN1965.1  	RENLDVSNCVIVSPDAGGAKR2ATAIADRLDLQFALIHK~ERPRPNEVSRM~VLVGSVKD
          	***   ..************* **::**:*:  ****** ****** *.** ****.*:*

MG07148.1 	KIAIL0VDDMADTCGT~LSKAAQTVKDHGAAEVVAIVTH1GI1LSGNAIENLNNSCLSKV
NCU06970.1	KIAIL~VDDMADTCGT~LVKAASVLKENGAKAVLALVTH~GI~LSGNAIENLNGSVLEAL
FG09299.1 	KVAIL~VDDMADTCGT0LAKAAETVHKHGASEVYAIVTH~GI~LSGKAIDTINSSVLSGL
AN1965.1  	KVAII~VDDMADTCGT~LVKAADTVMQHGAKEVNAIVVH~GI~LSGKAIDNVNNSCLKRI
          	*:**: ********** * ***..: .:**  * *:*.* ** ***:**:.:*.* *. :

MG07148.1 	VVTNVGTSNTRANHTGKANTVVCTRPQT~VPL~GNKVDR~CPKIRVIDVSATIAE0AIRR
NCU06970.1	ICTN-----------------------T~VPL~GDKIER0CPKIRVIDISPTIAE~AIRR
FG09299.1 	VVTN-----------------------T1VPL1GDKIER~CPKLKVIDVSGTLAE0AIRR
AN1965.1  	VVTN-----------------------T~VPH~KEKKEL~CDKIETIDISPTLAE~ACRR
          	: **                       * **   :* :  * *:..**:* *:** * **

MG07148.1 	THNGESVSHLFTHVPV
NCU06970.1	THNGESVSYLFNHAPV
FG09299.1 	THNGESVSYLFNNAPV
AN1965.1  	THNGESVSFLFSHTVA
          	********.**.:. .
```
